# Understanding Below-replacement Fertility in Kerala, India

**DOI:** 10.3329/jhpn.v28i4.6048

**Published:** 2010-08

**Authors:** P. Sadasivan Nair

**Affiliations:** Department of Population Studies, University of Botswana, Gaborone, Botswana

**Keywords:** Aging, Demographic transition, Fertility, Human development, Morbidity, Population growth, India

## Abstract

Kerala is well-known globally for the unprecedented fertility transition in the Indian subcontinent towards the end of the last century. The state has already reached below-replacement fertility level in the 1990s while the rest of India was experiencing high or mid-level fertility. With this backdrop, an attempt was made in this paper (a) to explore the plausible factors associated with sub-replacement fertility and consequent population momentum in Kerala and (b) to trace their socioeconomic and health policy implications. The underlying factors that led to the fertility transition was explored and discussed in some detail. An enhanced level of human development achieved during the last quarter of the 20^th^ century, mainly through developments in social and health sectors, is likely to be the main contributor. Unlike other states in India, there were historical factors as well that functioned as a catalyst for this, such as widespread education and women's empowerment. As an inevitable demographic impact, population growth due to momentum is expected to be very strong in Kerala with an age-structural transition favouring the old. The so-called ‘demographic dividend’ invoked by the increase of labour-force derived from the youth bulge in the age-structure is being lost in the state due to very limited capital investments and political will. Again, as a direct consequence of population growth, population density in Kerala will take a staggering level of 1,101 persons per sq km in 2026. The ill effects of environmental deterioration and consequent changes in morbidity patterns will have to be dealt with seriously. The very foundations of health policy needs revamping in the light of demographic changes associated with sub-replacement fertility. The tempo of population-ageing is very high in Kerala. The proportion of population aged 60+ years is likely to be 20% in 2026 whereas it will be around 12% only in India. The current level of social and health infrastructure in the state may not be sufficient to cope with the emerging demands of population-ageing since the financial and morbidity burdens of the elderly are already quite high. To conclude, Kerala portrays a typical case of the vagaries of the onset of sub-replacement fertility level in the absence of reasonable structural changes in the economic and health fronts.

## INTRODUCTION

Kerala, a southwestern state in India, has caught the imagination of social scientists world over in recent times as a demographic exception or a paradox. In 2001, the state had a total population of 31.8 million, which was 3.1% of the population of India (1). During the 1950s, the population growth rate in Kerala was one of the highest in India. However, by the 1970s, it began to fall significantly and subsequently became the lowest among the Indian states. During 1981-1991, the growth rate dropped to 14.3% and in the next decade, it dropped further to 9.4% whereas the corresponding figures for India were 23.9% and 21.3% (1). By 2006, Kerala had the lowest birth rate (around 14.7 per 1,000), the lowest death rate (around 6.8 per 1,000), the lowest infant mortality (13 per 1,000 livebirths), the highest life-expectancy at birth (73 years), and the highest literacy rate (91%) in India. It attained replacement fertility level (total fertility rate=2.1) in around 1987 and is currently experiencing sub-replacement fertility level. Thus, among the major states in India, Kerala has pioneered in completing the demographic transition during the last quarter of the 20^th^ century.

The case of Kerala is rather exceptional and even puzzling due to the fact that the correlates or antecedents of the demographic transition achieved have no resemblance with that of Europe. Much of the demographic transition in the West was an integral part of a development phase during which economic growth fostered material aspirations and improvements in living conditions (2). When the fertility and mortality transitions were so rapid in Kerala during the last quarter of the 20^th^ century, its economic growth trajectory was marked by low per-capita income and high rate of unemployment characteristically shared by many poor regions in the developing world. The state had a very weak industrial base and a stagnant agricultural sector with relatively low scope for labour absorption. In 1980-1981, Kerala was ranked eighth in terms of per-capita income among the Indian states, and its rank declined by one point in the next decade. Before 1987-1988, the economy of Kerala was characterized by near stagnation of major economic indicators. During this phase, the annual growth rate of net domestic product in Kerala was too low to cover even the population growth rate while, at the all-India level, it was 1.53% above the population growth rate (3). Nonetheless, Kerala has pioneered in achieving the highest levels of social and demographic development in India during the same period without recording corresponding economic growth. The adult literacy rate in Kerala was 90.9% in 2001 while, in India, it was only 65.4%. The rate of female literacy in Kerala was 87.9% while that in India was only 54.3% (1). Further, almost 100% of school-age population and the youth are literate. Kerala is again unique in India with a sex ratio favouring females, i.e. male-female ratio: 0.9:1 in Kerala in 2001 and 1.1:1 in India.

With this backdrop, an attempt was made in this paper, first, to explore the plausible factors or antecedents associated with sub-replacement fertility level and consequent population momentum in Kerala, and, second, to trace their socioeconomic and health implications for the state.

## MATERIALS AND METHODS

Data for the study were derived mainly from the Sample Registration System published by the census authorities in India from 1981 through 2007. Further, the author's previous work on the estimation of population momentum and age-structural transition of India, including ageing, was used. Various published materials on relevant historical factors, socioeconomic and health changes pertaining to Kerala were also used extensively.

The methodology used for the analysis included trend analysis, pattern recognition, and content analysis.

## RESULTS

### Fertility transition: main factors

Table 1 shows the trends in crude birth rate, total fertility rate, crude death rate, and infant mortality rate in India and Kerala in the recent past. Transition of consistent fertility decline in Kerala began much before the 1970s when several states in India just commenced significant fertility declines.

**Table 1.** Trends in total fertility rates, crude death rate, and infant mortality rate: comparison between India and Kerala, 1981-2007

Year	India	Kerala
Birth rate		
1981	33.7	26.8
1991	29.5	18.3
1994	28.7	17.4
2001	25.4	17.2
2005	23.8	15.0
2007	23.1	14.7
Death rate		
1981	12.5	6.6
1991	9.8	6.0
1994	9.3	6.1
2001	8.4	6.6
2005	7.6	6.4
2007	7.4	6.8
Infant mortality rate		
1981	110	37
1991	79	16
1994	74	16
2001	66	11
2005	58	14
2007	55	13
Total fertility rate		
1981	4.5	2.8
1991	3.6	1.8
1994	3.5	1.7
2001	3.2	1.9
2006 (NFHS-3)	2.7	1.9

Source: Registrar General of India. Reports of the sample registration system, 1981 to 2007. New Delhi: Registrar General of India, and International Institute for Population Sciences. National family health survey (NFHS-3), 2005-2006. Bombay: International Institute for Population Sciences, 2007 (4)

Several social scientists devoted their attention to explain this unprecedented phenomenon in Kerala. To cite a few, Ratcliffe attributed this decline to the structural changes in the political economy, land reforms, minimum wages in agriculture, and large public investments in primary and secondary education (5). Zachariah argued that the developments in public health and universal education over a long period, and increase in the number of surviving children together with parent's perceived higher cost of educating their children raised the cost of childrearing in Kerala and paved the way for the successful practice of family-planning methods (6). Further, higher literacy and educational level of women and rise in age-at-marriage played an important role in the improved healthcare of children within the family (7–10).

There was also a host of historical factors which remained, perhaps, as catalyst for the social change. The matrilineal system, followed by higher-caste Hindus, and the ruling class in Kerala and its dissemination effect played an important role in giving Keralite women a unique position they hold in India in terms of empowerment. [For a synthesis on this, see R. Jeffrey (11)]. The Maharajas (Kings) of Kerala from the 18^th^ to the mid-20^th^ century were known for their progressive ideas and reforms, which had far-reaching effects on the social milieu of Kerala. The Christian faith in Kerala is much older than in Europe or America and dates back to 52 AD when St. Thomas came to Kerala coast. Thereafter, several Christian missionaries also reached Kerala and built churches and schools. The modern educational institutions popularized by the Christian missionaries played a key role for the growth of literacy. Thus, Matriliny, Maharajas, and Missionaries have played a key role in the overall social transformation of Kerala historically. Triggered by high literacy, along with high unemployment among the educated, Keralites were the pioneers of Indian Diaspora.

The historical factors, in a way, prompted the successive democratically-elected governments in Kerala to continue to invest in education, health, and other social development sectors. Consequently, the literacy in the state increased continuously, and the health indicators showed much progress towards attaining the World Health Organizaiton's target of Health for All by 2000 AD. The overall social change brought in by these measures resulted in higher social equity and capillarity, higher age at marriage for girls and boys, and lower son preference. In other words, a much-needed social norm for smaller families began to emerge among all sections of Kerala society. These factors, along with intensive family-planning campaigns since the 1970s, resulted in higher rates of contraceptive prevalence. Kerala was the front-runner in many innovative family-planning campaigns in India. India relied so much in sterilization, a terminal method, in its so-called ‘cafeteria approach’ in family-planning service-delivery. In the 1970s, the ‘camp approach’ was introduced in popularizing vasectomy. In July 1971, a month-long vasectomy camp was organized in Ernakulam in Kerala where 62,913 operations were done using a simple surgical approach (12,13). This success story set a world record, and most states in India tried to replicate this, of course, without much success.

The large employment opportunities thrown open by the oil boom in the Gulf States around 1970 benefited millions of higher- and lesser-educated skilled labour-force in Kerala. The growing cash remittances from Kerala migrants buttressed the sagging economy, and the purchasing power of an average Keralite has increased substantially, although these were not visibly reflected in the official statistics concerning the state's gross domestic product or per-capita income.

All these factors contributed to the achievement of the highest level of human development for Kerala among the Indian states. The human development index value for Kerala in 2005 was 0.773, the highest among the states in India while that in India was 0.619 (4). The health and education indices in Kerala stood at 0.827 and 0.930 respectively whereas the income index was only 0.562 which shows a relatively higher development of health and education sectors and with a low profile of the economic front. The high level of human development and the already-evolved small family norm in the state had complimentary relationships. Demographic history is rampant with examples of non-reversal of the small family norm once achieved. The higher level of contraception, along with higher age-at-marriage, functioned as intermediate or proximate variables which led to lower fertility in Kerala.

### Population momentum

Kerala reached the net reproduction rate=1 in 1987 which implied a total fertility rate of 2.1. The net reproduction rate, a measure of the average number of daughters who will be born to women adjusting for their mortality from the time of their birth, of unity is analogous to just replacing mothers in the population and hence leads to the exact replacement of population in the long run (14–16). Even when the total fertility rate declines to replacement level, there is a lag period before the rate of natural increase declines to zero which can otherwise be termed as the growth potential left after reaching the replacement-level fertility. This is because people who have already been born when fertility was still high build ‘momentum’ into the population, which is referred to as population momentum. In other words, children outnumber parents in a growing population. Consequently, the number of potential parents in the next generation will be larger than at present (17). It is due to this seemingly strange demographic phenomenon that, despite attaining replacement-level fertility, the population of Kerala is still growing with a natural increase of 8.6 per 1,000 people, and it will take several years to reach zero population growth.

In an earlier work, Nair and Nair have estimated the quantum of population momentum for India and Kerala (18) using the generalized method of estimation for any observed population (19). It is estimated as the ratio of the ultimate stationary population-size to the initial observed size. The initial population is projected in five-year leaps for 150 years; after that the population becomes virtually stationary. We used the component method in estimating the stationary population as it provides information on the age-specific changes, which enables the estimation of age-specific momentum as well. Table 2 shows the summary estimates of momentum of population growth in Kerala and in India (figures in parentheses refer to the years of achieving replacement-level fertility).

**Table 2.** Momentum of population growth in India and Kerala

Area	NRR	Momentum
Kerala (1987)	1.00	1.59
India (2019)	1.01	1.34

NRR=Net reproduction rate

As Table 2 shows, while Kerala has the potential to add 59% to the population from the time of replacement-level fertility to zero population growth, India (which is expected to reach replacement-level fertility by 2019) has a further growth potential of 34% only.

Thus, the momentum of population growth in Kerala has relatively been enormous. This is due to the faster decline in fertility and mortality in Kerala in the recent past.

### Implications

The momentum-invoked demographic aspects with far-reaching implications in development planning of any country would be (a) population-size, including density, (b) age-structural transition, and (c) population-ageing.

### Population density

Table 3 presents the change in population-size and density during the next two decades in Kerala, along with corresponding projected values for India as a whole. Thanks to the tremendous inbuilt growth of population, density in Kerala will take a staggering level of more than 1,000 persons per sq km during 2021 itself and 1,101 in 2026 whereas the corresponding figures for India would be 453 and 472 respectively.

**Table 3.** Momentum-related population growth in Kerala vis-a-vis projected population of India

Population and density	2001		2021		2026	
	Kerala	India	Kerala	India	Kerala	India
Population (million)	31.8	1,039.7	39.6	1,347.7	40.7	1,406.2
Population density (sq km)	860	350	1,018	453	1,101	472

The resource-crunched state of Kerala looks already overcrowded even now, and the increase in density can have far-reaching consequences in the areas of environmental health, public-health infrastructure needs, housing, employment, etc. Environmental pollution is incredibly high even today. For instance, the quality of water and air has deteriorated tremendously in recent years. Kerala is a land of rivers, and the major water-quality problem is bacteriological pollution due to dumping of solid wastes, bathing, and discharge of effluents. Again, the chloride concentration of >250 mb/L was detected in the well-water samples in Kerala. The fluoride content was observed to be beyond the permissible limit of 1 mg/L. Faecal contamination is present in 90% of drinking-water wells (20). Similarly, the level of air pollution is also quite high. Vehicles are mainly responsible for the deterioration of air quality. Kerala recorded an astonishing increase of 200% in the number of vehicles during the 1975-2002 period. Personal transport vehicles constitute 72% of the vehicle population in the state. Two-wheelers that emit carbon monoxide at a higher level accounted for 77% of personal transport vehicles in the state (21). The ill effects of this environmental deterioration and consequent changes in morbidity patterns will have to be dealt with by the policy-makers on a war footing. The national policy on industrial location, which lacks pragmatism and vision, also adds to the vagaries of deterioration of environmental health. Despite the fact that the state has already reached the final stages of epidemiological transition, there were several outbreaks of hitherto unknown virus**-** related infectious and other diseases, such as dengue fever, *chikun* guinea, and leptospyrosis in recent years in Kerala. The health administration in the state was found to be in total disarray to manage this situation. Health-policy formulations in the state cannot afford to ignore these developments invoked largely by the momentum-related population growth and density.

### Age-structural transition

Age-structural transition is a direct consequence of fertility declines often mediated by shifts in the patterns of survivorship and migration flows. Kerala is in the second stage of age-structural transition while most Indian states and sub-Saharan countries are in the first stage. Since 1991, a speedy age-structural transition is underway in Kerala. Due to the continued flow of younger birth-cohorts from the large reservoir of couples in the reproductive age-groups, the younger age-structure did persist until 2001. As shown in the figure, the proportion of young dependants (age 0-14 years) started falling since then, and in 2026, the proportion will be 18.2%, i.e. a reduction of 30% since 2001 (22).

The reduction in the young population produces a wave effect in the intermediate age-groups that form the labour-force. Here, the proportion of population aged 15-59 years was 61% in 1991, which has grown to 64% in 2001. A significant reduction is expected in this age-group only after 2021 when the proportion of 60+ years is expected to increase substantially. The large volume of labour-migration flows mainly to the Gulf States has mediated the proportion of labour-force in Kerala. The proportion of elderly (60+ years) has been increasing linearly since the 1970s but the pace will be quite high from 2011 onwards when the third phase of age-structural transition ushers in.

**Fig.**
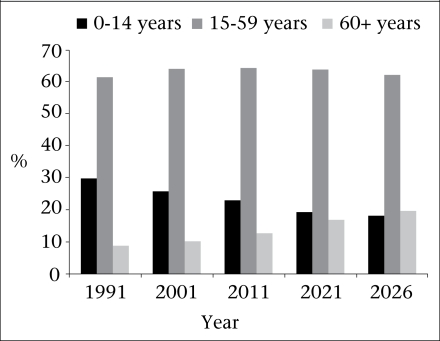
Age-structural transition, Kerala, 1991-2026

### Demographic bonus

The proportional reduction of the young population is good news for a state like Kerala, for it paves the way for reduced investments in social sectors, such as primary education and child health. Again, it triggers a wave effect later when the proportion of economically-active age-groups is bulged, resulting in the large flows of young workers into the labour market. As the figure shows, 64% of the population was in the labour-force in 1991, which is likely to increase to 68% in 2026. Both these aspects, invoked by the age-structural transition, brings in the ‘demographic bonus’ or ‘window of opportunity’ for the state. However, this will have positive outcomes only if there is capital investment and creation of job opportunities. If this population wave is not responded to in this way, this will be not merely an opportunity lost but will result in increased fiscal burdens and disinvestment in social welfare necessary to sustain young un- or underemployed (23). Opportunities are, thus, potential risks as well. Some authors have extensively studied this phenomenon recently (24–29), and it is encouraging to note that the state government, of late, started investing in tertiary sector (especially service), and employment opportunities are being created, although its impact is yet to be assessed.

### Population-ageing

Between the onset of replacement fertility and the advent of zero natural increase, a typical population will tend to become greyer, its median age will rise, and the proportion of the elderly will also rise. As younger cohorts move up the age pyramid, the middle and older age-groups are expanded in particular, and population-ageing is a direct consequence of momentum. Population momentum has been shown to be linearly related to ageing (30). Age-specific momentum shows that the momentum is much greater in the older age-groups (19). Among the Indian states, Kerala is the front-runner in this aspect. The proportion of the elderly (60+ years) increased from 5.8% in 1961 to 10.2% in 2001 and is expected to reach nearly 20% in 2026—a level currently observed in many developed countries of the West. For India as a whole, the population of 60+ years was around 7% in 2001 which is expected to rise to a modest 8% in 2011 and 11.6% in 2026 (31). While the total population is expected to grow on a slower pace (0.7%), the growth of elderly will be quite spectacular (4.9%) from 2011 onwards. Similarly, the median age of the population is expected to increase from 28.5% in 2001 to 38.5% in 2026. The index of ageing (the proportion of population aged 60+ years to the population aged 0-14 years) estimated was 13.7 in 1961, which is expected to increase to 108% in 2026. All these indicators of ageing point towards the accelerated ageing process currently underway in the state.

What are the policy-relevant effects of ageing in Kerala? By 2021, a 60-year old male is likely to survive for 21 years and that of a 60-year old female by 23 years. Obviously, widowhood rates are quite high. The number of widows per 100 widowers was about 800 in 2001 and is likely to increase to more than 1,000 by 2026. While half of elderly males were working, only 10% of elderly women were working in 1981. They were mostly engaged in agriculture where there is no retirement age. The traditional family support for the elderly is slowly disappearing and hence the need for a vibrant social security system. The state administration with its limited resources may not be in a position to envisage and implement such schemes for the fast-growing elderly population in Kerala.

Apart from the financial support required for their living, the disease burden of the elderly are also of great concern. The most significant aspect of epidemiological transition is seen in the morbidity status in the state when the health status of the aged shows a transition from communicable diseases to non-communicable diseases. The prevalence of chronic diseases, such as cardiovascular diseases, cancer, diabetes, and chronic lung diseases, is reported to be the highest in the state. Further, the disease-specific prevalence of morbidity among the elderly indicates that the most prevalent diseases are hypertension, both among males and females (total 14.1%: males 11.1% and females 16.6%), followed by disorders of joints and bones (total 11.3%: males 8.2% and females 13.8%), diabetes mellitus (total 9.9%: males 11.1% and females 8.9%), and asthma (total 5.7%: males 7.3% and females 4.3%). The healthcare expenditure for this segment of population is, therefore, bound to increase drastically over the next quarter of the century and beyond. Is the state prepared for this eventuality? There is no universal health-insurance scheme in the country as of now.

Regarding the availability of medical and paramedical manpower, Kerala's position is above the national level. The number of available hospital beds per 1,000 people is 3.44 in Kerala in 2001 while the recommendation of the World Health Organization is 32 beds. The current levels of the health infrastructure is definitely not sufficient to take care of the changing morbidity patterns. When there is potential for the population to increase by about 59% in Kerala in the coming decades, massive public investment is needed to cope up with the emerging situations. Apparently, the policy-makers and planners are not yet caught up with this situation, although social scientists and public-health professionals were engaged in issuing warning-signals at least since the last decade (33). It is worth noting here that even in a socially-advanced state like Kerala, geriatrics has not developed as a branch of medicare even in major hospitals.

Some authors have noted that Kerala has the highest morbidity rates (34) in the country which may not be realistic. This phenomenon is attributable to the changing perceptions of illness and health due to high literacy, the ageing of population, and declining case-fatality rates. It may be added that the high literacy, accessibility of healthcare providers, and, above all, the rights-oriented perception of public utility services of the common men in Kerala are other reasons for the reported high morbidity. In any case, the inevitable increase in the burden of morbidity on the healthcare system is bound to increase in the state with ageing of population.

Further, the social and emotional aspects of the aged population, especially of aged females, are also worthwhile concerns. The changing age-structure due to momentum will favour a higher old-age dependency ratio. In the light of family-structure transition underway in the state and a high level of emigration of adult population in search of jobs, the elderly are forced to be alone. As Golini states, the challenge will be to find very quickly and progressively the right formulae to manage the ageing process (35). This is the real revolution in the age-structure of a population, a very new phenomenon in the history of humankind and, perhaps, the most important demographic development of the 21^th^ century.

## DISCUSSION

Demographic transition in Kerala in the 20^th^ century defies conventional wisdom among policy-makers and social scientists in that it was not economic development induced as witnessed in the West in the late 19^th^ and early 20^th^ centuries. In a way, the experience in Kerala brings forth adequate raw materials for yet another theoretical formulation in the explanation of fertility transition in developing countries. It may be noted that one of the main weaknesses of the demographic transition theory is that it does not posit the threshold level of socioeconomic development required for a sustained fertility transition. The experience in Kerala shows that demographic transition is possible even in the absence of significant economic development. Kerala could achieve it through an enhanced level of human development brought in mainly through developments in social and health sectors.

Fertility in Kerala has declined to below-replacement level in the 1990s while the rest of India was still grappling with high or medium levels of fertility. The immediate demographic consequence of this phenomenon is the growth potential implied due to population momentum. It is expected to be very high in Kerala as it would be witnessing a massive increase in numbers (59%) in its transition to stationary or zero population growth compared to any other state in India. This implies that the population of Kerala will continue to grow with an age-structural transition favouring the old. The proportional reduction of young population and its wave effect on the labour-force due to higher past fertility have already brought in the ‘demographic dividend’ for the state. The state is, thus, experiencing the demographic dividend phase of a ‘youth bulge’ in labour-force, and it will continue the trend for some more time. If this population wave or ‘window of opportunity’ is not fruitfully responded with adequate policy changes in capital investments and creation of more and more jobs, this will be an opportunity lost and massive unemployment will follow. Opportunities are, thus, potential risks as well.

What are the socioeconomic and environmental implications of this momentum-induced population growth in Kerala? The density of population in Kerala will take a staggering level of more than 1,000 persons per sq km during 2021, and it is estimated to reach 1,101 in 2026. This can have far-reaching consequences in the areas of environmental health, public-health infrastructure needs, shelter, employment, etc. in a state which is already overcrowded. The changes in morbidity patterns will have to be dealt with by the policy-makers. The impact of the inevitable increase in the burden of morbidity on the healthcare system is likely to be very high in Kerala.

The tempo of population-ageing is quite high in Kerala. The proportion of the elderly (60+ years) was 10.2% in 2001 and is expected to reach nearly 20% in 2026. The median age of the population is expected to increase from 28.5 years in 2001 to 38.5 years in 2026. What are the policy-relevant effects of ageing in Kerala? Obviously, widowhood rates are expected to increase since the life-expectancy of female is higher as seen elsewhere. This means that the proportion of females far outweigh their counterparts in the aged segment of population in the state, and most of these females are widows, and the trend is likely to continue. While half of the elderly males were working, only 10% of the elderly women were working in 1981. Hence, the economic status of the widows is quite deplorable even today which is fuelled by the absence of family support and any worthwhile social security system in the country. This unfortunate scenario is quite likely to worsen further. Again, the ageing of population can result in reduced savings and investments due to the rising burden of deceases. The prevalence of chronic diseases, such as cardiovascular diseases, cancer, diabetes, and lung diseases, is also reported to be the highest in the state. Therefore, healthcare expenditure for this segment of population will increase drastically over the coming years. Even in a socially-advanced state like Kerala, geriatrics has not developed as a branch of medicare. Apart from governmental support, intergenerational economic support, especially from working sons, is also crucial here. If needed, government legislation is called for in ensuring the family support for the elderly as seen in some East Asian countries

To conclude, the experience in Kerala vividly portrays a typical case study of the vagaries of the onset of drastic fertility transition in the absence of a threshold level of the much-needed structural changes in the socioeconomic and health fronts.

## ACKNOWLEDGEMENTS

The author is grateful to the anonymous referees for their valuable comments for the improvement of this paper.
